# Is Male Infertility an Early Warning of More Serious Diseases?

**DOI:** 10.1210/endocr/bqaf187

**Published:** 2026-01-05

**Authors:** Dolores J Lamb

**Affiliations:** Childrens Mercy Hospital Department of Surgery/Division of Pediatric Urology and Children's Mercy Research Institute, Kansas City, MO 64108, USA; University of Missouri—School of Medicine, Kansas City, MO 64112, USA; Kansas University Medical Center University, Kansas City, KS 66160, USA

**Keywords:** male infertility, health risks, cancer, spermatogenic failure

## Abstract

About 12% of couples worldwide are infertile. Male factor infertility causes or is contributory to a couple's ability to conceive in approximately 50% of cases. Evidence has emerged that infertile men have poor overall health and increased morbidity and mortality, yet the causes for this are poorly understood. Although these men may appear healthy, research shows that they can harbor a wide variety of systemic diseases and illnesses that may share common links with the causes of their infertility. In fact, as semen parameters decline, their risks of several health conditions increase. In the early 1990s to the present, studies revealed that 1% to 6% of unselected infertile men seeking clinical evaluation have significant and (sometimes) life-threatening pathologies ranging from endocrine abnormalities to malignancies, developmental anomalies, and genetic diseases. Yet, despite this knowledge, for couples seeking treatment of their infertility, the female partner undergoes extensive clinical evaluation but the male partner frequently is only asked to provide a specimen for a routine semen analysis. This review focuses on the current understanding of the association of the genetic causes of male infertility and a multitude of diseases that affect these men’s overall health and their increased risk of mortality.

Infertility is a devastating disease for couples who expect to experience biological parenthood. They face the unexpected disruption of their life plan, as well as a significant financial cost associated with the treatment of this disease, in addition to emotional costs. According to the National Institutes of Health’s Eunice Kennedy Shriver National Institute for Child Health and Human Development (1), about one-third of infertility results from a female reproductive issue and one-third from a male reproductive issue, and in one-third of the couples, both partners contribute to the infertility or there are unknown factors that underlie the infertility. In this paper, the focus is on infertile men and their poorer overall health and increased risk of morbidity and mortality when compared with fertile men and the causes of these health risks.

Infertility presents a significant risk for the development of other diseases. Yet (in contrast to their female partners who usually undergo routine general health evaluations and gynecologic examinations), the male partners in an infertile couple are rarely examined for general health problems and for causes of their infertility. Significant medical pathologies, in particular malignancies (Tables 1 and 2) are present in 1% to 6% of patients presenting for a male infertility evaluation [reviewed in (9, 10, 95, 96)], as well as endocrine abnormalities, malignancies, cardiac and immune dysfunctions, genetic and karyotypic abnormalities and genitourinary birth defects (2, 3, 20, 23, 78, 79, 97‐102). Yet frequently, infertile men are not clinically evaluated when infertile couples are treated by in vitro fertilization ± intracytoplasmic sperm injection. Knowledge of the increased health risks of the infertile male points to the need for andrologic consultation when an infertile couple seeks treatment at an assisted reproductive technology clinic (4, 4, 103‐105). If properly evaluated, preferably by a fellowship-trained andrologist, men could be advised of the potential health risks associated with their infertility. Earlier intervention may improve their health outcomes. This review discusses the evidence that infertile men, in general, have increased morbidity, mortality, and systemic disease and higher overall health risks associated with their infertility. This paper is a narrative review of the literature concerning the association of male infertility with increased health risks of serious diseases with a focus on the underlying genetic causes common to male infertility and a multitude of other diseases.

**Table 1.** Causes of male infertility, cancer, and gene defects

Type of male infertility	Malignancies in infertile men	Gene defects	Gene-specific malignancies	Refs.
**All**	All or not specified	**Mismatch repair** **Lynch syndrome** *MLH1* *MSH2* *PMS1* *MGMT*	Colorectal cancer, Endometrium, Ovary, Stomach, Small Bowel, Urinary Tract, Biliary Tract, rain, glioblastoma, skin, pancreas, and prostate	(2‐33)
	Invasive germ cell tumors Testis cancer High-grade prostate cancer Bladder cancer Non-Hodgkin lymphoma Leukemia Thyroid cancer Melanoma	**Homologous Recombination** **Double-strand break repair** *MSH5* *RAD51B* *BRCA2* *LIG4* *BRIP1* *DMC1*	Prostate cancer Ovarian cancer Breast cancer Lung cancer Hematologic Non-Hodgkin lymphoma Leukemia Thyroid cancer	(7, 9, 10, 15‐17, 19, 24, 27, 34‐52)
		**Nucleotide excision repair base Excision repair** *POLB* *XPA*		
Abnormal semen parameters	Not specified Testis cancer Peritoneum	*CATSPER1* *CATSPER2*	Colon cancer Pancreatic cancer	(53, 54)
Spermatogenic failure (NOA)	Not specified	*MLH1*	Colorectal cancer Stomach cancer Pancreatic cancer Hepatobiliary tract cancer Brain cancer Sebaceous carcinoma Stomach cancer	(26‐33, 40, 49‐51, 55‐66)
NOA's family members	Bone and joint cancer Soft tissue and uterine cancer Thyroid cancer Hodgkin lymphomas	*MSH2* *MSH5* (see above)	Ovarian cancer, small intestine urinary tract	(67)
Oligozoo spermia	Testis cancer			(8, 11, 13, 16, 68, 69)
Oligozoosper mic's family members	Colon cancer Bone and joint cancer Testis cancer Esophageal cancer			(67)
Teratozoospermia MMAF	Colon cancer	*CATSPER* *CATSPERE*		(8, 13, 16, 68‐70)
Globozoospermia	Testis cancer	*DYP19L2* *SPATA16*		(71)
Asthenozoo spermia	Testis cancer	*DNAH1* *DNAH9*		(8, 13, 16, 68, 69)
Primary ciliary dyskinesia	Cancers of the brain, skin, gastrointestinal system, genitourinary system, thyroid, and sarcomas			(72)
Oligoasthenoteratozoo-spermia Asthenotera-tozoospermia	Testis cancer			(8, 13, 16, 68, 69)
Cryptorchidism	Testis cancer	*E2F1* *CRKL* *VAMP7*	Testis cancer Breast cancer Melanoma Lung cancer Liver hepatocellular carcinoma Pancreatic cancer Endometrial cancer Head and neck tumors Renal cancer Ovarian cancer Glioblastoma Colon cancer Liver cancer Bladder	(73‐77)

Abbreviations: MMAF, multiple morphological abnormalities of sperm flagella; NOA, nonobstructive azoospermia.

**Table 2.** Male infertility and its association with nonmalignant diseases and increased mortality

Type of male infertility	Cardiovascular diseases	Endocrine disorders, diabetes mellitus, male hypogonadism	Immune disorders	Mortality	Others	Gene defects	Ref.
All	Cardiovascular disease Congestive heart failure	Male hypogonadism Low testosterone	Rheumatoid arthritis Graves' disease Multiple sclerosis Thyroiditis Immuno-deficiency syndrome	Increased risk	Klinefelter syndrome Cryptorchidism Hypospadias Small testes Micropenis Gynecomastia	*MSH5*	(9, 57, 78‐83)
Abnormal semen parameters				Increased risk			(82)
Spermatogenic failure (NOA)	Cardiovascular disease Congestive heart failure	Male hypogonadism	Rheumatoid arthritis Graves' disease Multiple sclerosis Thyroiditis Immuno-deficiency syndrome	Highest risk	Klinefelter syndrome Cryptorchidism and/or hypospadias DiGeorge syndrome 16p11.2 microdeletion/ microduplica-tion syndrome	*MSH5*	(83‐85)
Oligozoospermia	Cardiovascular disease Congestive heart failure	Male hypogonadism	Rheumatoid arthritis Graves' disease Multiple sclerosis Thyroiditis Immuno-deficiency syndrome	Increased risk		*MSH5*	(83)
Teratozoospermia Globozoospermia MMAF						*CFAP43* *DNAH1* *CFAP44* *DNAH9*	(86‐88)
Asthenozoo-spermia Primary ciliary dyskinesia	Dextrocardia				Rhinosinusitis Bronchial sepsis Situs invs heterotaxy	*DNAH5, DNAH11CCDC39CCDC40* *ODAD1 ODAD2* *ODAD3* *ODAD4* *NNE8* *DNAL1* *SPAG1* *ZMYND10* and many more	(89‐91)
Oligoasthenotera-tozoospermia Asthenoterato-zoospermia			Multiple sclerosis		Deafness-infertility syndrome	*CATSPER1-4, CATSPHERE*	(53, 54, 92, 93)
Varicocele	Hyperlipidemia Vascular disease	Diabetes Low testosterone Male hypogonadism					(92, 94)

Abbreviations: MMAF, multiple morphological abnormalities of sperm flagella; NOA, nonobstructive azoospermia.

## General Health Concerns and Overall Mortality Are Increased in Infertile Men

Surprisingly, infertile men are at increased risk of many systemic illnesses, which can engender significant morbidity and mortality (Table 2). The Charlson comorbidity index (CCI) is a collection of 19 weighted parameters that can influence morbidity and mortality. These parameters include conditions such as diabetes and associated complications, congestive heart failure, liver disease, renal disease, chronic pulmonary disease, peripheral vascular disease, metastatic tumors, AIDS, and several blood cancers. Men visiting a fertility clinic who undergo a routine semen analysis (specifically semen volume, sperm concentration, motility, count, and morphology) have higher CCI scores, which portend an increased overall mortality risk (79). The lower the sperm count (oligozoospermia, nonobstructive azoospermia), the higher the CCI score, and men with higher sperm count had decreased mortality rates (lower CCI score). A decrease in the overall health status of men with primary infertility was shown in a retrospective study of their CCI scores in nearly 10% of infertile males in a 10-year time frame after their initial evaluation (80) with cancer, cardiovascular diseases, and diabetes mellitus being the most common ailments.

Immune disorders are more prevalent in infertile men (78) [hazard ratio (HR) 3.11], in particular rheumatoid arthritis (HR 1.29), Graves' disease (HR 1.46), thyroiditis (HR 1.60), and multiple sclerosis (HR 1.91) (9). These immunological disorders are associated with mutations in *MSH5,* a gene that encodes a protein involved in meiotic recombination during spermatogenesis and also with class-switch recombination within the call II region of the major histocompatibility complex region (57, 81). Men with multiple sclerosis have reduced sperm count, motility, and morphology (92). This led Eisenberg and colleagues to propose that infertility may reflect an early presentation of subclinical systemic autoimmune disease with infertility being the first obvious phenotype presented (78).

The increased risk of cardiovascular disease, including ischemic heart disease and diabetes in infertile males, may result to some extent from hypogonadism (106). Men with low circulating levels of testosterone (<213 ng/dL) have increased all-cause mortality. This is due in part to men with Klinefelter syndrome (46,XXY-XXXXY male), a common cause of nonobstructive azoospermia (NOA) (11%). The syndrome is characterized by tall stature with long limbs (due to extra copies of the *SHOX* gene on the sex chromosomes) and hypogonadism (84). The affected males may have cryptorchidism, micropenis, and hypospadias with small testes with hyalinization, fibrosis and azoospermia, infertility, low testosterone, and gynecomastia. Klinefelter men display significant phenotypic heterogeneity, and since clinicians are often unaware of this condition, the vast majority of patients go undiagnosed (84, 85).

Men with even lower testosterone (<153 ng/dL) have an even higher risk for cardiovascular death (107). A U-shaped association with all-cause and cardiovascular disease mortality was seen with low and very high levels of dihydrotestosterone. Higher SHBG concentrations are also associated with all-cause mortality (107).

Varicoceles are associated with vascular disease and a higher risk of diabetes and hyperlipidemia (94). An increased risk of other chronic medical conditions, such as alcohol abuse (HR 1.48, 95% CI 1.07-2.05), obesity, and drug abuse (HR 1.67, 95% CI 1.06-2.63) are also present in infertile men, as compared with men who only received infertility testing (79). Confounding this observation is the fact that illicit drug use is an important cause of infertility. Illicit drugs that have a negative effect on fertility include commonly used agents such as anabolic-androgenic steroids, cocaine, marijuana (feminizing-causing gynecomastia and spermatogenic problems), opioids, and methamphetamines [reviewed in (108, 109)].

Infertile men have a higher risk of mortality (99). In a study of nearly 135 000 infertile men and over 242 000 controls over a time-period of 3.6 and 3.1 years, respectively, infertile men had a higher risk of death (HR 1.42, 95% CI 1.27-1.60), and men with spermatogenic failure (no sperm in the ejaculate; nonazoospermia) had a significantly increased risk of death (HR 2.01, 95% CI 1.60-2.53) with a higher trend for men with lower than normal sperm concentration (oligozoospermia) (HR 1.17, 95% CI 0.92-1.49) compared to controls. Cardiovascular and malignant diseases did not account for the higher risk of death for infertile men, so the underlying causes of this risk remain poorly understood (99). A diagnosis of male infertility can elicit significant stress for the patient, resulting in poor lifestyle choices and increased mental angst. However, regardless of the inclusion of age or prevalent disease in the analyses, the men with spermatogenic failure (no sperm in the ejaculate) have the highest risk of death. Retrospective studies in Scandinavia, some with data collected over a longer time, had similar findings, with 1 long-term follow-up study reporting that the positive association of infertility and mortality could be explained by a cancer diagnosis prior to their infertility assessment (11).

While these findings are concerning, they must be put in perspective, as the overall risk is small and the overall mortality rates were lower than those reported for the US general population at large (99), perhaps because healthier men may be the ones who choose to become fathers and who may avoid dangerous situations, unintentional injuries, or perhaps suicide.

## Malignancies and Their Link to Genes Involved in Spermatogenesis or Sperm Function

In the 1980s, male infertility was first reported to be associated with testicular cancer. In this initial study, carcinoma in situ was present in a subset of these infertile men, the majority of whom later went on to develop invasive germ cell tumors (12). Subsequently, Walsh and colleagues (5, 13, 14) evaluated data on 22 562 partners of infertile couples and showed the risk of testis cancer was nearly 3-fold higher in infertile men compared with normal men. Many retrospective studies confirmed these results, showing significantly increased risks of testis cancer for infertile men (6, 14‐17, 110), 1 of which reported a 20-fold increased risk (see (9)). First-degree relatives of these infertile men have a 52% higher risk of testis cancer than the general population, suggesting a genetic cause (7).

The reasons testis cancer is associated with male infertility are not clearly understood. Abnormal semen parameters are associated with testis cancer. Jacobsen et al evaluated more than 32 000 men who had a semen analysis over a 30-year period and found a 1.6-fold increased risk of testis cancer, as well as cancers of the peritoneum and other digestive organs in men with lower sperm count (8, 11), consistent with the other studies described previously.

Defective spermatogenesis may result from not only spermatogenic failure resulting in oligozoospermia or azoospermia but also from defects in meiosis and spermiogenesis (the process of the haploid round spermatid undergoing nuclear condensation, formation of the flagella, and acrosome reaction to differentiate into a spermatozoa). Men with oligozoospermia (low concentration), teratozoospermia (poor morphology), or asthenozoospermia (motility defects) alone or in combination have a higher risk of testis cancer with highly significant HRs of up to 11.9 depending on the spermatogenesis anomaly present (8, 13, 16, 68, 69).

### Asthenozoospermia and Teratozoospermia and Cancer Risk

Among the functional sperm anomalies causing morphologic, count, and motility sperm defects, asthenozoospermic sperm (viable sperm with poor or absent motility) are commonly seen in the in vitro fertilization laboratory. Primary ciliary dyskinesia (PCD) is an autosomal recessive condition that causes male infertility due to immotile sperm (89, 111). Individuals with PCD also may have chronic rhinosinusitis, dextrocardia, and chronic bronchial sepsis due to disruption of the spermatozoa flagellar tail and other cilia structures, which have 9 + 2 (9 peripheral and 2 central) microtubular pair structures (89). These structures are connected with a nexin link, dynein arms, and radiating spokes (112), all of which are needed for proper sperm flagella (tail) motility. Clinical genetic diagnostic laboratories routinely assess 42 to 90 or more different genes for damaging mutations out of at least 500 clinically validated genes (many for less common causes of PCD) of at least 2515 genes implicated (some not validated or with sufficient clinical evidence for diagnostic use) in this disease (90). Ciliary defects are linked to a variety of oncogenic processes, and some patients with PCD and other types of defective cilia/flagella have developed cancers of the brain, skin, gastrointestinal system, genitourinary system, and thyroid and sarcomas (72).

Multiple morphological abnormalities of sperm flagella (MMAF) are another category of sperm flagella defect that causes asthenoteratozoospermia (sperm with poor or no motility and morphologic anomalies of the tail). A common MMAF abnormality is the loss of the 2 central microtubular pairs with a 9 + 0 axonemal structure, as opposed to the traditional 9 + 2 structure (86) with damaging mutations in cilia and flagella associated protein 43 (*CFAP43*), dynein, axonemal, heavy chain 1 (*DNAH1*), and cilia and flagella associated protein 43 (*CFAP44*) genes accounting for about 70% of the cases of MMAF (86). Using RNA sequencing and a bioinformatics approach, *DNAH1* (downregulated) and *DNAH9* (upregulated) were identified in 2 of 4 key hub genes predicted to be tumor-promoting factors, reliable potential molecular biomarkers, and therapeutic targets for treatment of human head and neck squamous cell carcinoma patients with the worst outcomes (87). DNAH1 family member gene mutations contribute to chemotherapy response in gastric cancer patients, as compared to wild-type controls (88). While some preliminary data suggests that there is also a link between *CFAP44* and cancer, additional studies are required to define this relationship. In contrast, the role of these proteins in flagellar structure and function is known.

Mutations in the *CATSPER* family of genes (*CATSPER1-4*), which encode cation channels of sperm, are associated with both nonsyndromic (*CATSPER1*) and syndromic (*CATSPER2*) male infertility with the latter causing deafness-infertility syndrome. *CATSPER1* deficiency results in poor sperm motility, low semen volume and sperm count, and morphology defects. *CATSPER2* mutations affect sperm concentration, motility, viability, and morphology (53, 54). CATSPERE, which encodes CatSper channel auxiliary subunit epsilon, is associated with colorectal cancer risk and enhances colon cancer progression (70). Other studies suggest that CATSPER regulates the PI3K/AKT signaling pathway to control colon cancer cell growth (93).

Globozoospermia (sperm with a round head with a missing, atrophied, or misplaced acrosome) is a morphologic defect of the sperm head that prevents the sperm from penetrating the oocyte to achieve fertilization; intracytoplasmic sperm injection of globozoospermic sperm with egg activation using a calcium ionophore in in vitro fertilization rarely can achieve fertilization or a live birth (113). Globozoospermia is linked to a higher incidence of testis cancer, specifically, due to gene defects of 2 of the most common causes of globozoospermia: mutated *DYP19L2* and *SPATA16* (71).

### The Risk of Development of Other Malignancies in Infertile Men Is increased as Well

Infertile men have an increased risk of all cancers diagnosed after their evaluation for male infertility (2) (Table 1). A study of 76 000 infertile men used insurance claims to reveal that there were elevated rates of all cancer types (non-Hodgkin lymphoma in particular) when compared with controls (3). Other studies reveal that infertile men are at increased risk of bladder cancer, leukemia, and thyroid cancer [reviewed in (9)]. A meta-analysis of 168 327 men diagnosed with infertility and 2 252 806 men who were not infertile from 8 published cohort studies revealed a 1.4-fold increased risk of many cancers (pooled odds ratio = 1.43, 95% CI 1.25-1.64) in the infertile men. Of 3 individual malignancies (testis, prostate, and melanoma) analyzed in greater detail, the cancer risks were significantly higher in infertile men (18). Men with male factor infertility were 2.6 times more likely to be diagnosed with high-grade prostate cancer (18 , 20‐23 ), although there are conflicting studies (114). A recent pedigree examination of infertile men with NOA and severe oligozoospermia revealed that both cohorts had increased cancer risks that varied by the degree of spermatogenic failure. Increased risk of bone, joint, soft tissue, uterine, and thyroid cancers and Hodgkin lymphomas was observed among the azoospermic males' families, and among the severely oligozoospermic families, colon, bone and joint, and testis common risks were increased. A risk for esophageal cancer was also observed for the oligozoospermic families (67).

## The Role of Genes Required for Mitosis and Meiosis in Spermatogenesis and the Increased Risk of Malignancy in Infertile Men

### DNA Repair Deficiency Impacts Oncogenesis/Cancer, Male Infertility

**Figure 1.**
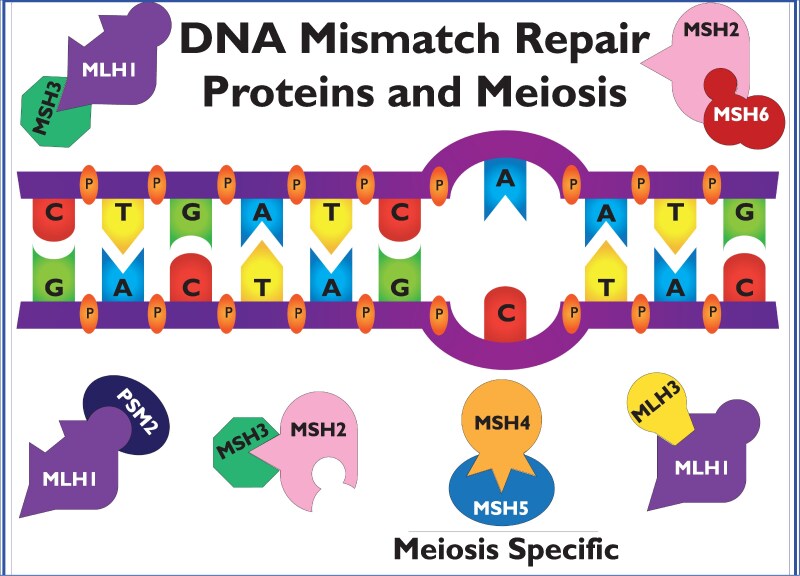
When DNA replication leads to de novo sequence variant mismatches, mismatch repair proteins work in tandem as heterodimers during mitosis and meiosis. Shown are the mismatch repair family of proteins that form the common heterodimers required for mismatch repair. Although structurally similar to the other mismatch repair proteins, MSH4 and MSH5 heterodimers working together are required for homologous recombination.

There are many types of DNA damage that are repaired by different mechanisms depending on the type of damage, including direct reversal (thymine dimers), base excision repair, nucleotide excision repair, mismatch repair (MMR), single-strand break repair, and double-strand break (DSB) repair, the most deleterious type of DNA damage. The MMR system is highly conserved across species and made up of families of proteins called MutS, MutL, and MutH that work together to recognize the mismatch, identify the site, and remove and repair the mismatch. In humans and mice, MSH1-6, MLH1 and MLH3, and PMS1 and PMS2 (Fig. 1) (obligatory for postmeiotic segregation) are MMR proteins homologous to bacterial MutS and MutL homologues. Deficiencies of MMR genes are associated with increased cancer risk because cells have a mutator phenotype and accumulate many unrepaired mutations, leading to malignancies.

**Figure 2.**
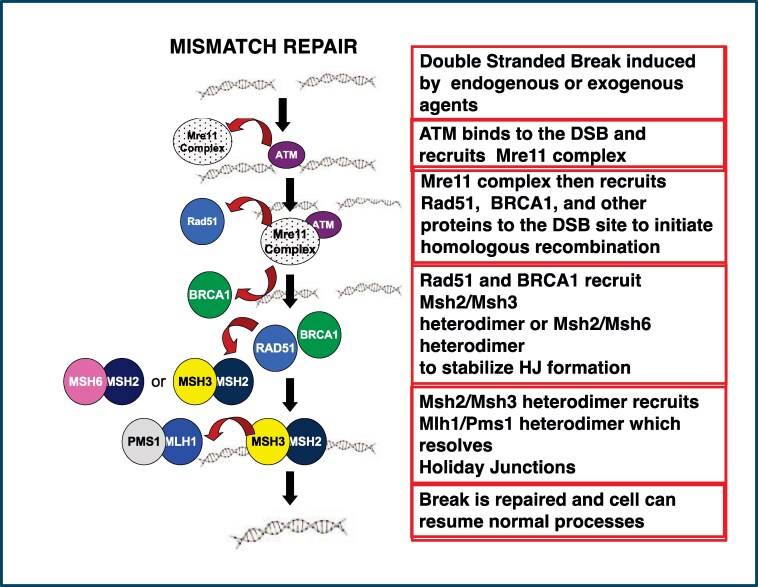
The process of mismatch repair showing the interactions of the proteins involved: mismatch repair depends not only on the proper formation of the different heterodimers during repair but also the function of other proteins in this process, such as ATM, BRCA1, RAD51, and the MRE complex in a highly regulated manner. When a double-strand break occurs in meiosis, the cell must recognize the damage; ATM binds and recruits the Mre11 complex, which in turn recruits RAD41 and BRCA1 to initiate homologous recombination, which in turn recruits MSH2/MSH3 or MSH2/MSH5 to stabilize the Holliday junction. Then MSH2/MSH3 recruits the MLH1/PMS1 heterodimer to resolve the Holliday junction, the break is repaired, and the cell resumes its normal function.

A brief review of these pathways is needed to fully understand the role of these proteins in both spermatogenesis and normal cell division (and infertility and oncogenesis) (Fig. 2). MMR deficiency is associated with an increased risk for colorectal cancer and cancers of the endometrium, ovary, stomach, small bowel, urinary tract, biliary tract, brain (usually glioblastoma), skin (sebaceous adenomas, sebaceous carcinomas, and keratoacanthomas), pancreas, and prostate and may result from a genetic disorder, Lynch syndrome (57, 115). Homologous recombination, 1 of 2 main types of DSB repair, is predominantly involved in meiosis during gametogenesis and allows immunoglobulin biosynthesis to create antibodies with different antigenic specificity, and nonhomologous end joining [DSB repair in somatic cells (116)]. Unrepaired or misrepaired DSBs may result in genomic instability, genetic aberrations, or cell death (Table 3). Homologous recombination requires that the cell recognizes the DSB and performs synthesis dependent on strand annealing, followed by break-induced replication. During the zygotene phase of meiosis, SPO11, a highly conserved topoisomerase, is necessary to initiate this process in a highly controlled induction of DSBs, to begin the recombination process. *Spo11-*deleted mouse models failed to form DSBs into homologs during spermatogenesis, resulting in defective synapsis, recombination, and ultimately meiotic arrest [reviewed in (117)]. MRE11 displaces SPO11 from the DSB site, causing a 3′ overhang and triggering the recruitment of RAD50 and NBS1 to repair the DNA ends for homologous recombination, resulting in the formation of double Holliday junctions and crossover products to repair the double-strand break (117) (Fig. 2).

**Table 3.** Function of MMR and consequences of deficiency

**MMR protein functions** To correct replication that escapes proofreading Required for homologous recombination during meiosis MMR improves replication fidelity 100- to 1000-fold	**Consequences of deficiencies** Elevated mutation rate Mutator phenotype Abnormal response to DNA damage due to alkylating agent exposure Increased cancer susceptibility Genomic instability Microsatellite instability Aneuploid gametes
**Causes of MMR deficiencies** Mutation Promoter hypermethylation (MLH1, MSH5) Altered expression of MMR genes	

In response to exposure of the cells to an alkylating agent, which causes extensive DNA damage, the cells should undergo apoptosis when the damage cannot be repaired. However, with MMR deficiency the cells continue to proliferate despite extensively damaged DNA. There is also microsatellite instability in the DNA, resulting in genomic instability. Aneuploid gametes may be formed as well as impaired gametogenesis. Finally, the cells may become senescent and fail to proliferate.

Abbreviations: MMR, mismatch repair.

### MMR Is Required during Mitosis and for Homologous Recombination during Meiosis

DNA repair protein MutS homologues recognize lesions in single-base pair mismatches, and MutL homologues coordinate downstream repair events. MSH2 forms a heterodimer with MSH6 and MSH3, whereas MLH1 heterodimerizes with PMS2, PMS1, and MLH3. MSH2-MSH6 and MSH2-MSH3 detect a set of single-base pair mismatches and insertion-deletion loops (Figs. 1 and 2). An unrelated MSH heterodimer (MSH4-MSH5) recognizes Holliday junctions (a unique DNA structure formed in homologous recombination) (Figs. 1 and 2). MSH4, MSH5, MLH1, and MLH3 are required for meiotic chromosome segregation, and MSH4-MSH5 and MLH1-MLH3 may interact during meiosis in ways that are independent of MMR (34) (Figs. 1 and 2). The MMR system also mediates G2-M checkpoint arrest and cell death after DNA damage (118). MMR/homologous recombination proteins and RAD51 are highly expressed in testes and are required for spermatogenesis (34‐37).

### The Maintenance of Genomic Integrity Is One of the Most Important Functions of Spermatogenesis

It is estimated that premeiotic germ cells in the male divide more than 1000 times prior to entering meiosis in a 50-year-old man (35). Evidence of strong mechanisms that account for nearly perfect germ-line DNA replication and maintenance is revealed by epidemiological studies showing a consistent measurable association with genetic defects in the offspring of older men (119‐124). Nevertheless, there is an astounding accuracy of replication in the germ cells. Replication is less efficient in somatic cells, which are vulnerable to carcinogenic mutation. This difference likely results from differences in DNA repair mechanisms between somatic and germ cells. In the germ cell, the homologous recombination system is used for DSB repair, as compared to the nonhomologous strand-joining system (which is far more error-prone) used by the somatic cells. At the start of homologous recombination, DSBs are introduced into each chromosome and then repaired by genetic exchange with allelic sequences resulting in physical linkages between pairs of homologues [reviewed in (38)]. The DNA MMR system and other DNA repair proteins, required for spermatogenesis, improve the fidelity of DNA replication by several orders of magnitude and are required for meiosis. The MMR pathway status affects mitosis and meiotic recombination, DNA-damage signaling, apoptosis, and cell-specific processes (microsatellite expansion, somatic hypermutation) (39). There is emerging evidence that compromised DNA repair, microsatellite and genomic instability, and a higher incidence of cancer/mortality are present in NOA men (Table 3).

Given the focus of this paper, the emphasis is on MMR and DSB repair for homologous recombination, both of which plays a key role in maintaining genomic stability through correct replication errors and improving the fidelity of replication. We hypothesized that these men had a systemic problem that underlies their infertility and affects spermatogenesis and that DNA repair deficiencies and genomic instability might be an unrecognized etiology of their infertility.

### MMR Deficiencies Are Associated with Spermatogenic Failure

In 2003, our laboratory realized that not only did mouse models with deletion of MMR genes encoding MSH2 and MLH1 have genomic instability, which predisposed the mice to cancer, but these male mice also exhibited spermatogenic defects similar to those testicular histopathologies seen in the biopsies of some human testicular failure patients (24). When MMR processes are deficient, this results in an increase in spontaneous mutation rate, checkpoint failure, and microsatellite DNA and chromosome instability (25) (Table 3). Microsatellites are repetitive segments of DNA scattered in the genome (coding and noncoding regions) that are used for linkage analysis, paternity testing, and forensic analyses. An in vitro diagnostic test intended for the qualitative detection of a novel panel of 7 monomorphic biomarkers in malignancies was used for detection of microsatellite status and fragment length in the blood and testis DNA of 40 men with NOA and 20 fertile control men. Microsatellite instability (MSI) is considered high when 2 or more mutant markers are present and stable when 1 or fewer are present. MSI and DNA MMR protein defects are present in some NOA men. Most of these MSI-H men had a Sertoli cell-only phenotype and DNA repair protein defects (*P* < .01 and *P* < .05, respectively); 10.5% of the Sertoli cell-only patients presented high MSI, with multiple loci analyzed showing instability (24). Conversely, high MSI was not observed in any controls or maturation arrest patients. It was present in 8.3% of hypospermatogenesis patients. Immunohistochemistry showed that MSI and MLH1 and MSH2 MMR protein defects, such as diminished or absent expression or protein mislocalization in the biopsies and cultured cells, were present in most men with spermatogenic failure in this small cohort (24). Importantly, these MMR protein defects were not germ cell-specific. The germ cells (if present), as well as the somatic cells, showed the same immunohistochemical identified abnormalities (24, 117). This study provided evidence of a previously unrecognized etiology of testicular failure associated with cancer predisposition (24).

Single-nucleotide polymorphisms (SNPs) and damaging mutations of MMR pathway genes were identified in mouse models and/or human patients with spermatogenic failure (26‐33, 65, 66). During spermatogenesis, the MutL homologues (MLH1, MLH3, and PMS2) and MutS homologues (MSH4 and MSH5) are required for gametogenesis. The loss of either MSH4 or MSH5 results in the failure of prophase I progression during meiosis, the failure of homologous synapsis, cell death resulting in germ cell loss with many seminiferous tubules showing only a single layer of spermatogonia and Sertoli cells, and Leydig cell hyperplasia (40, 49).

### The Role of MSH5 in Cancer and Infertility

MSH5 plays an important role in mitotic DNA recombinational repair but does not play a role in MMR despite being a member of this structural protein family. MSH5, once thought to be meiosis-specific, also participates in the cellular response to DNA damage, albeit not through MMR. Although *MSH5* is usually considered to encode a protein required for meiosis, independent approaches all point to *MSH5* as a susceptibility gene for cancer. A cross-cancer analysis of about 60 000 SNPs at 229 DNA repair gene regions across 5 cancer sites (breast, colon, lung, ovary, and prostate) revealed that *MSH5* is 1 of 3 known susceptibility DNA repair genes (*RAD51B* and *BRCA2* are among the others) with a highly significant association with lung and weaker associations with colon and ovarian cancers (50). Similarly, *RAD51B* and *MSH5* are associated with prostate cancer (60). The potentially damaging *MSH5* P768S SNP shows significant allelic association with chronic lymphocytic leukemia, non-Hodgkin lymphoma, and other hematologic malignancies (51, 52). The registry study of cancer risk in men with NOA published by our group did not have sufficient numbers of men to stratify those individuals by malignancy (3). Thus, malignancies in NOA may account for a significant percentage of the cancers found in the general population of infertile men.

In men with NOA, *MSH5* gene variants or damaging mutations can be associated with increased DNA fragmentation (damage) in normozoospermic men in the former and spermatogenic failure/meiotic arrest in the latter situations (55, 56). *MSH5* deficiency is implicated in the development of malignancies and immune disease, as well as infertility due to spermatogenic failure and increased DNA fragmentation in their sperm (2, 3, 5, 8, 14, 20, 24, 39, 50, 51, 57‐64, 78, 81, 125‐142) (Table 2). MMR and homologous recombination deficiency are associated with infertility in mouse models of targeted gene deletion, human premature ovarian failure, and meiotic deficiency (50‐52, 58‐62, 81, 91, 126‐128, 130, 138, 142, 143).

### Genitourinary Birth Defects Represent a Well-known Risk Factor for Male Infertility, Spermatogenic Failure, and a Plethora of Health Risks

Cryptorchidism (failure of 1 or both of the testes to descend into the scrotum during fetal development) is among the most frequently observed birth defects in full-term males, affecting about 3% of full-term newborn boys (144), and is a known risk factor for testis cancer, as well as male infertility due to spermatogenic failure. Testis cancer may result from a systemic impact on spermatogenesis, rather than simply due to the effect of increased scrotal temperature on the cryptorchid testis. Semen abnormalities, predominantly sperm concentration defects, occur in 30% of men with unilateral cryptorchidism and in 80% with bilateral cryptorchidism (23). Even after orchidopexy (surgically placing the testis into the scrotum), which includes permanent anchoring of the testis into the scrotum, many men continue to have spermatogenic failure (145). Recently identified causes of human genitourinary birth defects are associated with health risks, including infertility. Several of these findings are summarized next.

### E2F Transcription Factor-1 Microdeletions and Microduplications Underlie a Genetic Risk Factor for Testicular Cancer, Cryptorchidism, and Male Infertility

*E2F1* microdeletions or microduplications are associated with testicular germ cell tumors, cryptorchidism, and/or male infertility/spermatogenic failure in humans (146‐150). A microduplication at 20q11.2 was identified in men with NOA (147). *E2F1* plays multiple roles in regulating DNA synthesis, repair, cell proliferation, and differentiation, while in other cellular settings, this protein activates autophagy. Mouse models of targeted deletion and overexpression showed that overexpression caused male infertility due to testicular atrophy from germ cell depletion due to apoptosis (151, 152). The mouse models with loss of *E2F1* had increased incidence of tumors and testicular atrophy with aging (151, 153). In humans, about 7% of men tested with NOA resulting from cryptorchidism (corrected with an orchidopexy) had copy number gains or losses of *E2F1*. NOA men with microdeletions encompassing *E2F1* had a Sertoli cell-only phenotype with or without maturation arrest and Leydig cell hyperplasia or maturation arrest and hypospermatogenesis. Men with microduplications showed similar histopathologies, and they exhibited altered *E2F1* expression (146). *E2F1* also plays a key role in the regulation of testicular descent and spermatogenesis through its impact on WNT4 signaling (150). *E2F1* overexpression may also impact testicular descent through its repression of androgen receptor transcription (154‐157), as it does in prostate cancer cells. Copy number variants (microdeletions or microduplications) of *E2F1* may impact spermatogenesis and cryptorchidism by increasing germ cell susceptibility to heat stress (149). These microdeletions and microduplications of *E2F1* identified in NOA males with spermatogenic failure and cryptorchidism (147, 150), which impact both DNA damage repair and cancer development in general, are also a risk factor for testis cancer (148, 149), as well as for breast cancer and melanoma (158, 159) (Table 2).

### Chromosomal Microdeletions in the DiGeorge Syndrome Region (del22q11.2) Encompassing CRK-like Proto-oncogene Cause Micropenis and Cryptorchidism

About 1.4% of nonsyndromic males (and 31% of known DiGeorge syndrome males) have microdeletions encompassing *CRKL* with congenital anomalies of the kidney and urinary tract such as renal agenesis, multicystic dysplastic kidney, hydronephrosis, vesicoureteral reflux, irregular bladder, and voiding dysfunction. Four percent to 6% of DiGeorge syndrome males have cryptorchidism, and 4% to 8% have hypospadias and/or micropenis (160‐162). Additional abnormalities can also be present, including endocrine abnormalities (9). Loss of *CRKL* increased the risk of meningomyelocele by 23-fold, which was mediated by the common 22q11.2 deletion encompassing this key neural tube-expressed gene (163). Although fertility studies in humans with *CRKL* deficiency were not performed, studies in mouse models revealed cryptorchidism and reduced fecundity, reduced sperm concentration, abnormal testicular histology, and smaller testis size (161).

### Congenital Bilateral Absence of the Vas Deferens: A Genital Form of Cystic Fibrosis

Congenital bilateral absence of the vas deferens (CBAVD) is a genital form of cystic fibrosis, and 95% of men with cystic fibrosis have CBAVD. It is present in approximately 1% to 2% of infertile men and 6% of men with obstructive azoospermia. Damaging mutations in the gene that causes CBAVD (*CFTR*) account for nearly all patients with isolated CBAVD (164, 165). The gene is large, and to date, there are over 2000 mutations identified. Some mutations are severe and others mild based on their developmental and functional effects (165). The combination of mutations determines severity; 2 severe mutations in *CFTR* cause cystic fibrosis, and 2 mild or 1 mild and 1 severe can cause CBAVD. Routine genetic testing finds at minimum 1 mutation in about 80% of all cases and identifies less than 50% of patients who have 2 mutations (1 each in both alleles; compound heterozygotes). Accordingly, many patients with isolated CBAVD are misdiagnosed as negative because the routine tests cannot distinguish a carrier (where a second mutation is not found) from a patient incorrectly diagnosed as CFTR negative due to too few variants tested in the diagnostic panel, incomplete gene coverage, or determining whether other causative genes are mutated (165). There is an additional gene [adhesion G protein-coupled receptor G2 (*ADGRG2*)] (166) associated with CBAVD that is X-linked and is an epididymal (as well as gastrointestinal tract) expressed transmembrane protein (166). Deficiencies of *ADGRG2* were noted in a subset of patients with an obstructive azoospermia phenotype together with unilateral renal agenesis (96).

### Many Other Gene Defects Related to Genitourinary Anomalies Are Associated with Health Risks, Comorbidities, and Male Infertility

In general, endocrinopathies are a surprisingly infrequent cause of male infertility (with estimates ranging from 1-5%). Given the dependence of the male reproductive system on a functional hypothalamic-pituitary-gonadal axis, there is a complex crosstalk of hormones that are required for normal testicular function. Endocrinopathies affecting male reproductive function include hypogonadotropic hypogonadism (Kallman syndrome) caused by an absence of gonadotropin secretion due to a malfunction of the GnRH-secreting neurons. These anomalies result from damaging mutations affecting the migration of the GnRH-secreting neurons during development (*KAL1, KAL2*) and pituitary function (*GnRH11/GNRHR, PROK2, PROK2b*); the gonadotropin subunits for FSH and LH and their biosynthesis; the receptors in the testis for LH, FSH, and their downstream signaling pathway genes; as well as the genes required for steroid biosynthesis and steroid metabolism, steroid receptors (*AR, ESR1*), and their downstream signaling pathway genes. Hypergonadotropic hypogonadism due to Sertoli or Leydig cell dysfunction causes increased FSH or LH secretion together with low testosterone secretion; other anomalies include excesses of androgens, estrogens, or prolactin or abnormal insulin secretion (167, 168). Testosterone supplementation therapy and anabolic steroids will feed back to suppress LH production by the pituitary. All of these types of abnormalities have deleterious impacts on male infertility and genitourinary development (167, 169). It is also an issue for males on exogenous testosterone therapy or who take anabolic steroids or certain recreational drugs (108, 109).

### Microdeletion and Microduplication Structural Chromosomal Defects Impacting Androgen Receptor Action Causing Cryptorchidism and/or Hypospadias

Gene-dosage increases of the vesicle-associated membrane protein 7 SNARE gene, *VAMP7,* impacts estrogen action during development of the genitourinary system to potentiate the transcriptional activation of the estrogen receptor by increasing expression estrogen-responsive genes implicated in hypospadias. *VAMP7* overexpression also blunts androgen receptor action by targeting the ligand-bound receptor to the endosomes for degradation, resulting in less effective nuclear translocation of the androgen receptor to the nucleus (170), which was later confirmed by others (171). *VAMP7* is involved in exosome-, lysosome-, and autophagy-mediated secretion that is implicated in carcinogenesis and sarcoma progression, as well as neurodegeneration (172). Gene-dosage changes in *VAMP7* reported in the Database of Chromosomal Imbalance and Phenotype in Humans using Ensemble Resources reveal that other systemic symptoms include intellectual disability, facial dysmorphisms, autism, delayed speech, microcephaly, seizures, spasticity, and endocrinopathies [reviewed in (9, 96, 173)].

Similarly, copy number variants (microdeletions) at 16p11.2 cause loss of a gene, *KCTD13,* which encodes a substrate-specific adapter of a B-cell receptor complex (BTB-CUL3-RBX1) E3-ubiquitin-protein-ligase complex, which underlies cryptorchidism in 2.58% of males with cryptorchidism due to significantly decreased levels of nuclear androgen receptor and results in abnormal masculinization of the male genitourinary tract development (174) due to increased ubiquitination and degradation of the androgen receptor (175). Other gene defects impacting androgen action cause complete or partial androgen insensitivity syndrome, 5-α-reductase deficiency, persistent Mullerian duct syndrome, disorders of testosterone biosynthesis, resulting in a spectrum of phenotypic anomalies and in some cases increased risk of malignancies, hernias, stature, and endocrinopathies in addition to male infertility [see (9, 95)].

## Summary of Gene Defects in Male Infertility, Increased Health Risks, Morbidity, and Mortality

In conclusion, a subset of NOA men likely have a systemic problem affecting not only the spermatogenic cells but also the somatic cells. Proteins involved in mismatch and homologous recombination showed deficiencies due in part to gene mutation (or aberrant hypermethylation) in NOA men (41, 64, 176‐180). Genomic instability and subcellular mislocalization of the defective MMR protein is evident in some patients (24), as well as MMR gene mutation or hypermethylation and microsatellite instability (24). This is a systemic problem for NOA men, and these DNA repair deficiencies undoubtedly have adverse consequences for NOA men, similar to those observed in Lynch syndrome patients with MMR defects (57, 57, 181‐183). The genes most commonly mutated in the classic form of the disease are *MSH2, MLH1, PMS2, MSH6,* and *EPCAM6* with somatic microsatellite instability associated with female infertility and recurrent miscarriage [reviewed in (184)] and several different malignancies in men and women described earlier. In the future, additional research may reveal the impact of other DNA repair pathway genes on reproductive function in humans.

In addition to the DNA repair deficiencies associated with malignancies described here, a wide range of different types of genes impacting genitourinary development, spermatogenesis, and spermatid differentiation, as well as spermatozoa function, are also associated with malignancies; immune dysfunctions; other birth defects; and increased morbidity, mortality, and health risks ranging from cardiac and vascular abnormalities, intellectual disability and cognitive dysfunction, growth retardation and skeletal disorders, as well as respiratory diseases, including primary ciliary dyskinesia and cystic fibrosis.

## Conclusions

Given the higher risk of mortality that is present in infertile men, it is likely that more health risks will be uncovered as their overall health is more closely studied. In the future, once the etiologies that underlie men's infertility are more clearly understood, these patients could be advised of their potential health risks. For example, infertile men, especially NOA men, can be closely followed for cancer development. Earlier intervention may improve their outcomes.

## Data Availability

Data sharing is not applicable to this article as no datasets were generated or analyzed during the current study.
